# Viruses of Economic Impact on Tomato Crops in Mexico: From Diagnosis to Management—A Review

**DOI:** 10.3390/v14061251

**Published:** 2022-06-09

**Authors:** Raymundo Saúl García-Estrada, Alfredo Diaz-Lara, Vivian Hayde Aguilar-Molina, Juan Manuel Tovar-Pedraza

**Affiliations:** 1 Laboratorio de Fitopatología, Coordinación Regional Culiacán, Centro de Investigación en Alimentación y Desarrollo, Culiacán 80110, Mexico; rsgarcia@ciad.mx; 2 Tecnologico de Monterrey, School of Engineering and Sciences, Campus Queretaro, Santiago de Querétaro 76130, Mexico; diazlara@tec.mx (A.D.-L.); vivaguilar1105@gmail.com (V.H.A.-M.)

**Keywords:** *Solanum lycopersicum*, virus, symptoms, detection, distribution, transmission

## Abstract

Tomato is the most economically important vegetable crop worldwide and the second most important for Mexico. However, viral diseases are among the main limiting factors that affect the productivity of this crop, causing total losses in some cases. This review provides key information and findings on the symptoms, distribution, transmission, detection, and management of diseases caused by viruses of major importance in tomato crops in Mexico. Currently, about 25 viruses belonging to nine different families have been reported infecting tomato in Mexico, but not all of them cause economically significant diseases. Viruses of economic importance include tomato brown rugose fruit virus (ToBRFV), tomato spotted wilt virus (TSWV), tomato yellow leaf curl virus (TYLCV), pepino mosaic virus (PepMV), and tomato marchitez virus (ToMarV). The topics discussed here will provide updated information about the status of these plant viruses in Mexico as well as diverse management strategies that can be implemented according to the specific circumstances of each viral pathosystem. Additionally, a list of tomato-affecting viruses not present in Mexico that are continuous threats to the crop health is included.

## 1. Introduction

In Mexico, tomato (*Solanum lycopersicum* L.) production represented 25.4% of the total Mexican products that were exported in 2019, with an estimated value of USD 1908 million (Servicio de Información Agroalimentaria y Pesquera; https://www.gob.mx/siap, accessed on 10 December 2020), which positioned Mexico as the ninth largest producer and the major exporter of tomato worldwide, mainly for markets in the USA, Canada, Japan, and European countries. The main Mexican states that produce tomato are Sinaloa, San Luis Potosí, Michoacán, Jalisco, Zacatecas, Baja California Sur, Puebla, Morelos, Baja California, and Sonora (Servicio de Información Agroalimentaria y Pesquera; https://www.gob.mx/siap, accessed on 10 December 2020).

An integrated management of viral diseases in plants, including tomato, involves the combined use of all effective control measures to reduce economic losses. Strategies are applied in sequence or simultaneously, before or after transplantation or harvest, and are conditioned by the specific characteristics of each pathosystem [1]. At present, there are no chemical products (antiviral or viricide) available to control disease caused by plant viruses, and the most used strategy consists of controlling the source of inoculum and/or populations of insect vectors, but this practice has a limited rate of success. Therefore, the most efficient approach for protection against viruses remains the genetic resistance of host plants [2].

A deeper understanding of the epidemiology of viral diseases is crucial to improve current control techniques. Most viruses that cause diseases or epidemics in agricultural crops are transmitted by a vector (insects, mites, fungi, protozoan, nematodes, etc.), although there are exceptions, such as viruses in the genera *Tobamovirus* and *Potexvirus,* which are transmitted by contact. Therefore, the dynamics of the viral population should not be only considered in epidemiological analysis but also the vector–virus and host–virus interactions as well as the environmental conditions that affect the development of the disease [1].

There are several reported viruses in Mexico that are causing problems either in fields or greenhouses with tomato (Table 1). Nevertheless, certain viruses have gained greater attention than others due to their prevalence and the economic losses they have caused. Some of these are tomato brown rugose fruit virus (ToBRFV), tomato spotted wilt virus (TSWV), tomato yellow leaf curl virus (TYLCV), pepino mosaic virus (PepMV), and tomato marchitez virus (ToMarV). This manuscript seeks to provide a summary of the current status of these viruses in Mexico regarding symptomatology, distribution, host range, detection, and management.

## 2. Tomato Brown Rugose Fruit Virus

ToBRFV is a (+) single-stranded RNA virus and belongs to the genus *Tobamovirus* in the family *Virgaviridae*. The virus particles have a rigid rod shape, and its genome has a length of 6.2 to 6.4 kb, encoding four proteins: two replication-related proteins of 126 and 183 kDa resulting from the partial suppression of a stop codon; a 30 kDa movement protein (MP); and a 17.5 kDa coat protein (CP) [32,33,34].

**Figure 1:**
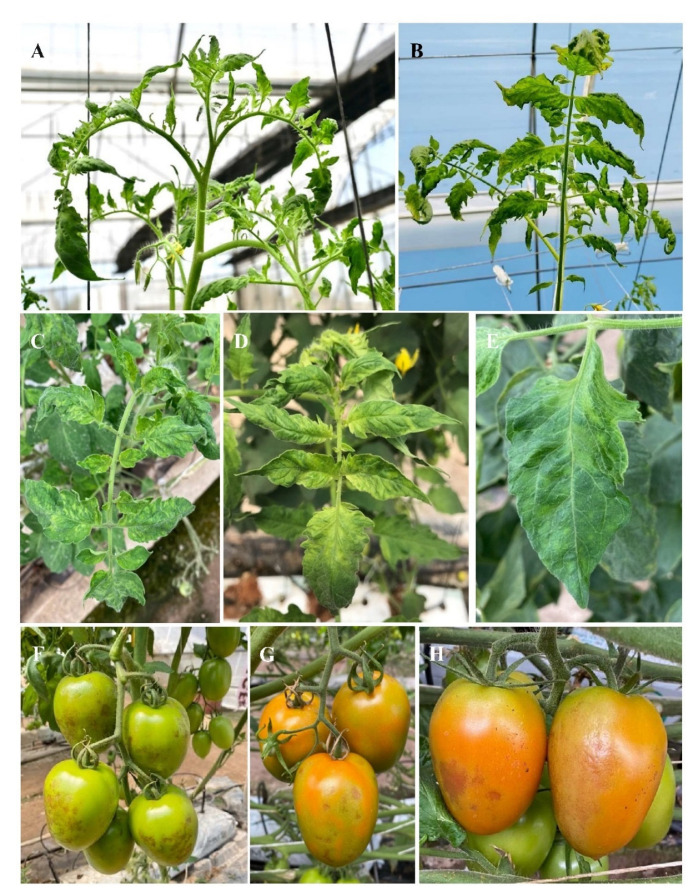
Symptoms caused by tomato brown rugose fruit virus on tomato plants. (**A**,**B**) Symptoms in apical growth point. (**C**,**D**) Leaflets with mosaic patterns and deformations. (**E**) Leaflets with mosaic patterns and a reduction in the leaf blade. (**F**–**H**) Fruits with brown spotting and necrosis in calyxes.

The symptoms caused by ToBRFV are observed as mosaic patterns on leaves and a distortion of the leaf blade, and on some occasions, calyxes may present necrosis (Figure 1A–E) [35]. Tomato fruits may exhibit brown spots, wrinkles, deformation, and irregular ripening (Figure 1F–H) [33,36,37]. Nevertheless, the presence of symptoms either on leaves or fruits will depend mainly in the response to the infection (susceptibility or tolerance) by different commercial tomato cultivars [32,33,38].

ToBRFV is widely spread across the world, with reports from the leading tomato-producing countries, such as Jordan [32], Israel [33], Mexico [18], USA [39], Germany [40], the United Kingdom [41], Italy [42], Palestine [43], Turkey [44], China [36], the Netherlands (National Plant Protection Organization; https://english.nvwa.nl, accessed on 1 June 2021), Greece [45], Egypt [46], Spain [47], Iran [48], Saudi Arabia [49], Norway [50], France [51], and Switzerland [52]; additionally, some suspicious cases have been reported in Chile, Ethiopia, and Sudan, but these remain to be confirmed [53]. In Mexico, ToBRFV was first reported in 2018 at Yurécuaro and Tanhuato, Michoacán [18], as well as in tomato produced at Ensenada, Baja California [19]; however, the virus spread rapidly and is now present in every tomato-producing area of the country [35].

ToBRFV is an emerging virus with the capacity to infect tomato cultivars harboring R genes *Tm-1*, *Tm-2*, or *Tm-2^2^*, which confer resistance to tobamoviruses [34]. Hence, the relevance of ToBRFV relies on the severe losses it might cause to tomato crops as a result of symptoms on fruit. Moreover, ToBRFV is of major concern for protected agriculture due to the number of plants that are grown (up to 50,000 plants per ha) as well as further handling during cultural practices that can result in additional spread within a facility [35].

The plants reported as natural hosts of ToBRFV are tomato and chili (*Capsicum annuum*). However, other experimental hosts have been described, including *Chenopodium amaranticolor*, *C. quinoa*, *C. giganteum*, *Chenopodiastrum murale*, *Nicotiana benthamiana*, *N. clevelandii*, *N. glutinosa*, *N. tabacum*, *N. sylvestris*, *Petunia hybrida*, and *Solanum nigrum* [33]. In Mexico, seedlings from different chili varieties were artificially inoculated, and defined symptoms were observed [54].

ToBRFV is transmitted either mechanically or by seed, as are the other tobamoviruses that infect tomato. Recently, [55,56] proved that ToBRFV is carried on the seed coat, sometimes in the endosperm but never in the embryo. Furthermore, studies have shown that transmission occurs through micro-wounds in seedlings developed during germination from infected seeds; the transmission rate between seed and seedling is 0.08% [56]. In [57], the authors indicated that the bumblebee *Bombus terrestris* may spread the virus.

Initial studies of ToBRFV implemented bio-indexing and electronic microscopy for its characterization [32,33]. By being considered an emerging viral disease in tomato, first reports of ToBRFV have become frequent across the world, which mostly involve molecular detection (RT-PCR). Consequently, there is a vast list of primers available for the specific detection of the virus. Real-time RT-PCR and reverse transcriptional loop-mediated isothermal amplification (RT-LAMP) have also been developed for its detection [42,58,59,60]. Multiple studies showed that ToBRFV can be successfully identified with next-generation sequencing (NGS) [33,57]. In addition, antibodies for ToBRFV are produced and commercialized and are employed in serological tests; however, cross-reactivity with other tobamoviruses is a possibility. Finally, a new detection method based on CRISPR/Cas technology has been recently described, which has the potential for use in the laboratory or field [61,62].

For the management of this disease, distinct strategies must be considered, such as the use of pathogen-free seeds, avoiding reusing substrates, the early elimination and removal of infected plants, the elimination of potential weed hosts located either inside or near the production area, crop rotation when possible, and avoiding the rotation of workers among greenhouses. Additionally, the cleaning and disinfection of work tools, machinery, hands, shoes, and the greenhouse structure are important. Although resistant cultivars with effective and durable R genes represent the most effective strategy for ToBRFV control, currently there are no commercially available resistant cultivars [63]. However, it was recently demonstrated that a quadruple knockout of *TOBAMOVIRUS MULTIPLICATION1* (*TOM1*) homologs in tomato confers a strong resistance to this virus. Therefore, tomato plants with strong resistance to tobamoviruses, including ToBRFV, can be generated by CRISPR/Cas9-mediated multiplexed genome editing [64].

## 3. Tomato Spotted Wilt Virus

TSWV belongs to the Orthotospovirus genus and the Tospoviridae family. Its particles are spherical and are surrounded by a membrane with a diameter of 80 to 120 nm. The membrane contains two glycoproteins (Gn and Gc) that form spicules over the surface and are required for the acquisition and transmission of the virus by thrip vectors. The genome of TSWV has ambisense/negative polarity and presents three segments of RNA that are designated according to their length: large (8.9 kb), medium (4.8 kb), and small (2.9 kb) [65]. Finally, each genomic RNA is encapsidated by multiple copies of the viral nucleocapsid (N) protein to form ribonucleoprotein structures also known as nucleocapsids [66].

**Figure 2:**
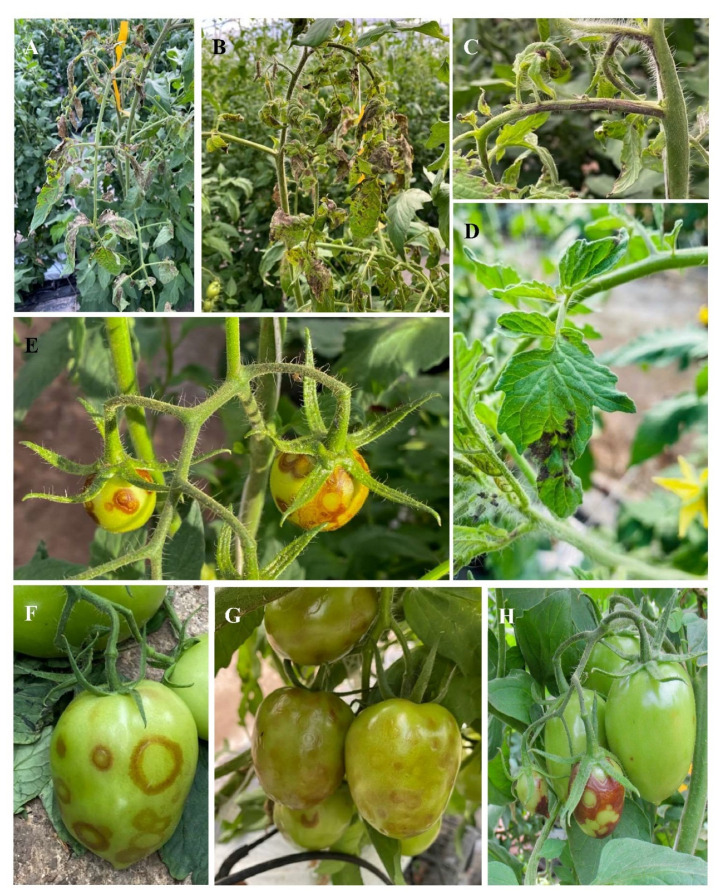
Symptoms caused by tomato spotted wilt virus on tomato plants. (**A**–**D**) Necrosis in stems, leaves, and flowers. (**E**–**H**) Fruits with concentric and necrotic rings.

The symptoms caused by TSWV in tomato vary according to the genotype of the plants and are more severe in plants that were infected young. Thus, young plants may exhibit stunting and necrotic spotting, mostly in buds or at the apical tissue of the plants, where leaves, stems, and flowers end up being affected (Figure 2A–D). In fruits, concentric and necrotic rings are observed as well as chlorosis and deformation (Figure 2E–H) [67].

TSWV is extensively spread around the world and is a virus with a presence in mild, tropical, and subtropical weather [65,68]. In Mexico, TSWV is usually found in the main tomato-producing states, including Sinaloa.

TSWV has been involved in severe outbreaks in tomato, causing a 42% reduction in productivity and about a 100% loss in the value of commercial tomato under field conditions [69]. In 2005, around USD 20 million were lost in the Central Valley of California due to a severe epidemic of TSWV and its vector [70].

This virus has one of the widest ranges of hosts among plant viruses. It can infect approximately 1100 plant species (including crops and weeds) from 85 botanic families. These include plants in the following taxonomic families: Amaranthaceae, Apiaceae, Asteraceae, Balsaminaceae, Begoniaceae, Brassicaceae, Campanulaceae, Caryophyllaceae, Chenopodiaceae, Convolvulaceae, Cucurbitaceae, Fabaceae, Geraniaceae, Lamiaceae, Malvaceae, Polygonaceae, Primulaceae, Ranunculaceae, Scrophulariaceae, Solanaceae, Verbenaceae, and Violaceae [68,71].

TSWV is transmitted in a propagative, circulative, and persistent manner by at least nine species of thrips (Thysanoptera: Thripidae) from the genera Frankliniella and Thrips, with F. occidentalis being the most efficient and important vector species in Mexico as a result of its wide range of hosts and distribution. Lastly, only the larval stage of the thrips (mainly the first instar) is the one that acquires the virus and transmits it to adult thrips, which are responsible for transmitting it to other tomato plants [72].

The molecular detection of this pathogen can be carried out using end-point or real-time RT-PCR and RT-LAMP [67]. Additionally, some protocols that include the use of immunocapture and microscopy have been reported [73]. Recent work implemented hyperspectral imaging for the detection of TSWV on infected plants before they started showing symptoms [74,75]. For practical purposes, under field or greenhouse conditions, commercial immunostrips for TSWV are usually used.

The management of TSWV involves more than just controlling the insect vector through the application of insecticides (chemical, biological, and botanical extracts). Thus, the combination of different strategies is required, such as resistant cultivars, the timing of planting dates, the application of resistance inducers, and the use of reflective plastics and sticky blue traps for thrips in addition to the control of weed hosts of the vector and the virus [68,76,77,78]. The most effective strategy for controlling orthotospoviruses in tomato is the generation of resistant cultivars. Currently, the Tsw and Sw5 genes are applicable for commercial resistance breeding against orthotospoviruses. The Tsw gene is highly specific and only confers resistance against TSWV isolates, while Sw5 confers a broad resistance against TSWV and various other orthotospoviruses [79].

## 4. Tomato Yellow Leaf Curl Virus

TYLCV is a single-stranded DNA virus that belongs to the *Begomovirus* genus (*Geminiviridae* family). Its monopartite genome consists of 2787 nucleotides, which are encapsidated by two incomplete icosahedrons, and contains two open reading frames (ORFs) in the sense orientation: V1 codes for CP and V2 codifies a MP-like protein with RNA-silencing suppression properties. In the antisense direction, the genome includes four ORFs: C1 encodes a protein associated with replication (Rep), C2 is a transcription activator protein, C3 is a replication enhancer protein, and C4 is a small protein embedded into Rep [80].

**Figure 3:**
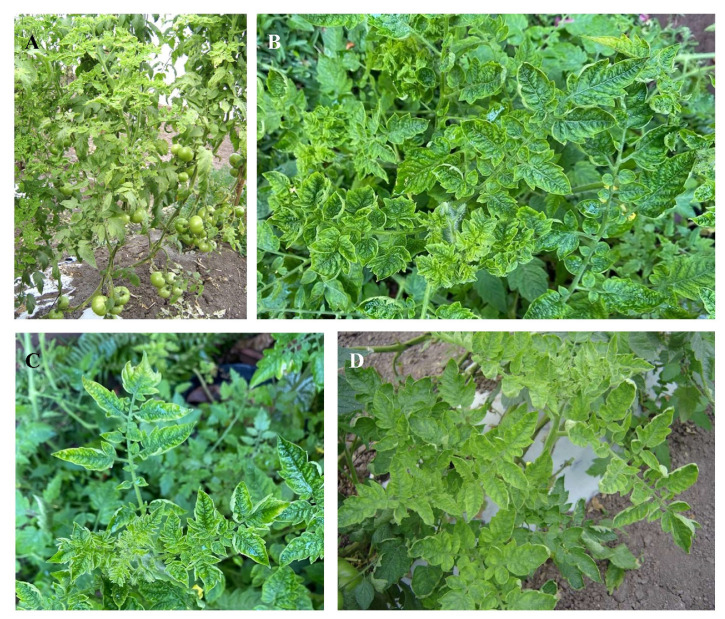
Symptoms caused by tomato yellow leaf curl virus on tomato plants. (**A**) Infected plants showing growth delay as well as yellowing and curling of the leaflet margins (spooning). (**B**–**D**) Leaflets of tomato leaves with curling and yellowing.

The symptoms caused by TYLCV are dwarfism, internode shortening, chlorosis, yellowing, and the curling of leaf margins (Figure 3A–D) [81]. Alternatively, fruits and flowers fall off and the plant’s growth stops [67].

TYLCV is widely distributed across the world, and it is present in the main tomato-producing areas, especially in those with subtropical and tropical climates [81,82]. In Mexico, the pathogen has been detected in tomato fields and greenhouses.

This virus causes an upward curling of leaves and is considered one of the pathogens with a higher dispersion and economic relevance to tomato production in tropical and subtropical regions across the world since it can cause losses of up to 100% [67,68,80]. TYLCV has a high mutation and genetic recombinant capacity Additionally, TYLCV is frequently identified in coinfections with other geminiviruses [83].

TYLCV presents a broad range of cultivated hosts, such as tomato, chili, beans (*Phaseolus vulgaris* L.), and tobacco (*N. tabacum* L.) as well as some ornamental plants, such as petunia (*Petunia* spp.) and lisianthus (*Eustoma grandiflorum* Shinn) [68,80]. Correspondingly, the virus has been detected in 49 types of weeds from the families Amaranthaceae, Chenopodiaceae, Asteraceae, Convolvulaceae, Brassicaceae, Euphorbiaceae, Geraniaceae, Leguminosae, Malvaceae, Orobanchaceae, Plantaginaceae, Primulaceae, Solanaceae, Apiaceae, and Urticaceae [84].

This virus is transmitted efficiently by whitefly (*Bemisia tabaci*, Hemiptera: Aleyrodidae). As the minimum acquisition period of the virus by the insect is between 15 and 30 min, the transmission is circulative and persistent [62].

PCR is the most used tool for the diagnosis of TYLCV, even though there are also tests based on rolling circle amplification (RCA), LAMP, and DNA hybridization [67,85]. As well as in other tomato viruses, NGS allows the identification and characterization of TYLCV isolates in infected plants [86]. Numerous serological techniques have been developed for the detection of this virus. Nevertheless, these detection methods present some inconveniences, as the sensitivity is not considered adequate for detection of all the virus variants [67].

Some efficient approaches for the management of the disease caused by TYLCV in tomato are maintaining control over the populations of the vector insect with the application of insecticides along with the elimination of alternative hosts for the virus, the implementation of yellow sticky traps, and barrier crops for the whitefly [68]. However, the most effective way to control TYLCV is breeding for resistance. Six resistance/tolerance genes (*Ty-1* to *Ty-6*) have been described, but the *Ty-1*, *Ty-2*, and *Ty-3* genes are widely used for tomato breeding [87,88].

## 5. Pepino Mosaic Virus

PepMV belongs to the *Potexvirus* genus and *Alphaflexiviridae* family. The virions are non-enveloped, filamentous, and flexible (470–580 nm with a diameter of 13 nm), and they contain chains of positive single-stranded RNA. Its genome, approximately 6.4 kb in length, includes five ORFs that encode a replication-associated protein of 164 kDa and three MPs of 26, 14, and 9 kDa (triple gene block) as well as a CP of 25 kDa [89].

**Figure 4:**
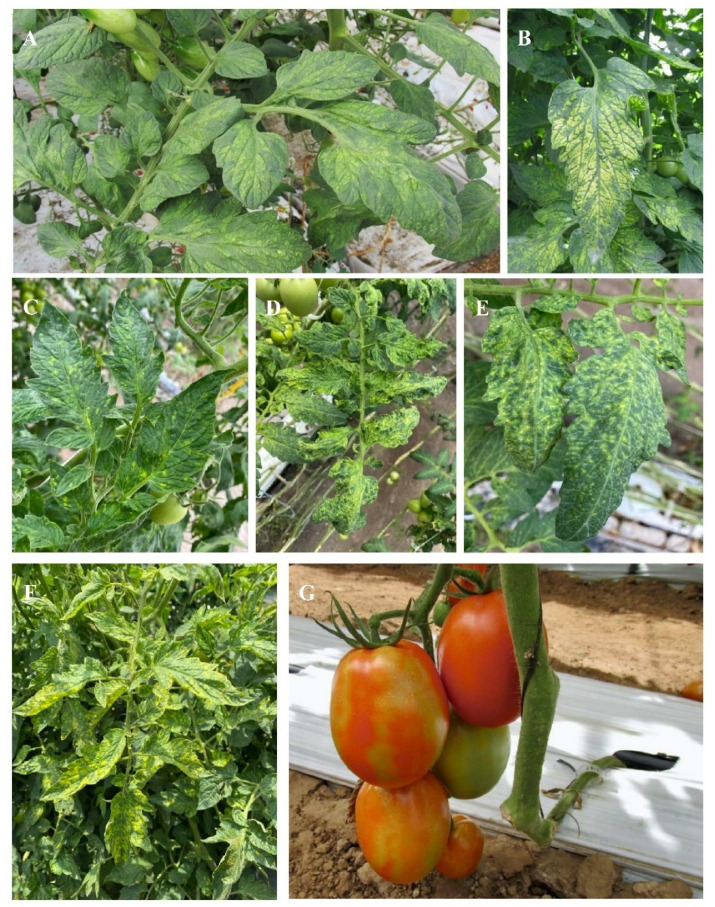
Symptoms caused by pepino mosaic virus on tomato plants. (**A**–**F**) Mosaic patterns and yellow spotting in leaves. (**G**) Fruits with yellow spots (mottle).

The expression of symptoms depends on the environmental conditions and the properties of the viral isolate. In tomato leaves, mosaic patterns, yellow angular spotting, distortion, and blisters are often observed (Figure 4A–F); meanwhile in fruits, irregular discoloration or a mottled pattern can be present (Figure 4G).

Since its report on tomato in Europe in 1999, PepMV has spread across the world in most tomato producing countries [68,89]. In Mexico, the presence of this virus was first confirmed in 2011, affecting tomato plants in a greenhouse located at Jocotitlan, State of Mexico [30]. However, the virus spread and is now present in all the main tomato-producing areas of the country, mainly in the central region.

To date, five strains of PepMV have been reported: the European (EU), Chilean (CH2), North American (US1/CH1), Peruvian (LP), and new Peruvian (PES) [90]. In the case of severe strains, the yield loss of commercial fruit can be up to 40%, and the effect of PepMV in the gross yield of fruit range from 5 to 10% [89].

Tomato is the most economically important host that is affected by PepMV, although the natural hosts of the virus are *S. muricatum*, *S. chilense*, *S. chmielewskii*, *S. parviflorum*, *S. peruvianum,* and *Ocimum basilicum.* In experimental inoculations, eggplant, potato, *N. benthamiana*, *D. stramonium*, *C. annuum*, *C. murale*, *Physalis floridana*, *Calystegia sepium*, *Diplotaxis erucoides*, *Heliotropium europaeum*, *Sonchus tenerrimus*, *Plantago afra,* and *Rumex* sp. were infected [91].

PepMV is mechanically transmitted by direct contact between plants as well as with contaminated tools, machinery, hands, and clothes, in which it could remain virulent for about 14 days. In addition, PepMV can be present for approximately four weeks in dry plant material and tomato roots [67]. The virus has a low rate of transmission (<2%) by seed, and can be spread by water, pollinator insects, and fungus *Olpidium virulentus* [89].

The first confirmed presence of PepMV in tomato was based on evidence generated by electron microscopy, bio-indexing, and RT-PCR [92]. In the case of RT-PCR, specific primers for PepMV or universal primers for *Potexvirus* viruses can be employed [93]. Polyclonal antibodies produced from the original isolate of PepMV and commercial antibodies are being used in ELISA, immunoelectron microscopy, and several serological methods [94]. Subsequently, analyses involving RT-LAMP and NGS were validated for the detection of PepMV [24]. For practical purposes, it is important to use virus-specific immunostrips for early confirmation of infection.

The most important strategies for the control of the disease caused by PepMV are the prevention of infection by strict hygiene measures and the chemical treatment of the seed. Cross protection can be effective but only under controlled circumstances and when a single PepMV isolate is dominant in a tomato production area [89]. Nowadays, there are no commercial resistant cultivars to PepMV; however, moderate resistance to the virus has been found in accessions of *S. peruvianum* and *S. chilense* [95]. On the other hand, the *Rx* gene has been shown to be active against PepMV, providing a source of resistance; however, some studies have indicated that the *Rx*-based resistance against PepMV in tomato may not be durable [96]. Lastly, tomato plants infected with PepMV at an early growth stage must be eliminated to diminish the rapid spread of the pathogen during cultural labor [68].

## 6. Tomato Marchitez Virus

ToMarV is a virus that consists of two molecules of positive single-stranded RNA. It belongs to the *Torradovirus* genus (*Secoviridae* family). RNA 1 is approximately 7 kb, containing one ORF that codifies for a polyprotein associated to replication. RNA 2 is approximately 5 kb and contains two ORFs. ORF1 codifies for a protein with unknown function, while ORF2 codes for MP and three viral CPs via a polyprotein. It is important to mention that even though tomato apex necrosis virus (ToANV), tomato chocolate virus (ToChV), tomato chocolate spot virus (ToChSV), and tomato necrotic dwarf virus (ToNDV) were proposed as separate species inside the *Torradovirus* genus, the comparisons between the nucleotide sequences and amino acids show high levels of identity with those of ToMarV. Hence, ToANV, ToChV, ToChSV, and ToNDV are actually considered to be isolates of ToMarV [22,97].

**Figure 5:**
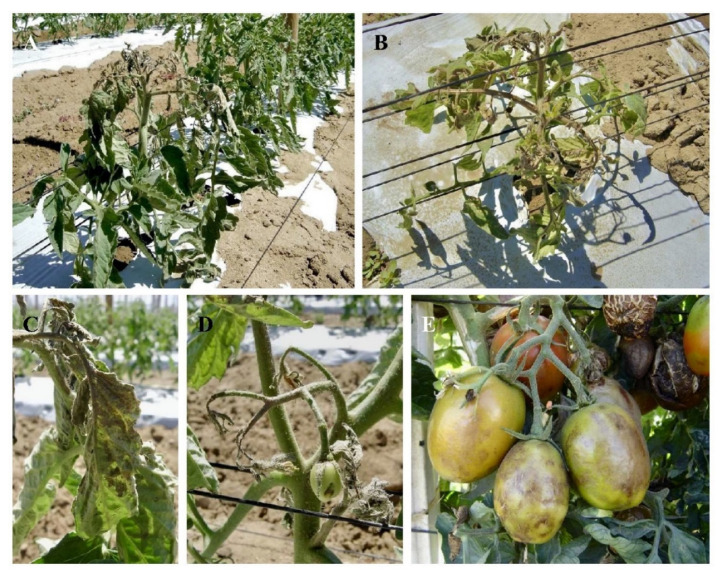
Symptoms caused by tomato marchitez virus on tomato plants. (**A**,**B**) Infected plants showing necrosis in the superior third. (**C**) Leaflet necrosis. (**D**) Flower necrosis. (**E**) Fruits with necrotic spotting.

The symptoms of ToMarV include necrosis at the growth points (shoot apex), resulting in a descending wilting (Figure 5A,B) [22]. The remaining older leaves sometimes turn necrotic too, but the necrosis rarely extends through the central stem. The necrosis of the individual leaves starts with little dark spots at the base of the leaflets, and eventually the spots merge and cover the whole base (Figure 5C) [21]. Other symptoms include growth delay, necrosis in flowers, necrotic spotting, and corky in fruits (Figure 5D,E) [21,23].

Since its first report in 2007 in Mexico, ToMarV has been identified in additional countries, including Guatemala and the USA [21]; in the particular case of Mexico, the virus has predominately been identified in tomato plants localized in the states of Sinaloa, Sonora, and Baja California Sur.

The damage in tomato seedlings can be up to 100%, and in fruit producing plants damages can be up to 60%. Moreover, ToMarV has been found to cause severe symptoms of yellow mosaic patterns, the upward curling of leaves, wrinkles, and growth delay in chili pepper plants in Sinaloa [98].

The only known natural hosts of ToMarV are tomato and pepper (*C. annuum*) [97], but the following plants have been artificially infected: *C. quinoa*, *N. glutinosa, N. benthamiana, N. occidentalis*, *N. hesperis*, *N. tabacum*, *N. rustica*, *P. floridana*, *P. phyladelphica*, *Datura stramonium*, *N. clevelandii*, *N. megalosiphon*, *S. nigrum*, and *Catharanthus roseus* [21,22,97].

The viral particles of ToMarV are retained inside the stylet of whitefly (Hemiptera: Aleyrodidae) and transmitted in a semi-persistent manner by *B. tabaci, Trialeurodes vaporarorium,* and *T. abutilonea* [99]. Furthermore, the virus can be mechanically transmitted in chili plants, but the transmission efficiency is much lower than in tomato [98]. In the case of transmission by seed, there is no information available for this virus.

ToMarV can be transmitted by mechanical inoculation to indicator plants, which express typical symptoms, such as mottle and foliar necrosis [22]. In 2008, the first virions of ToMarV from a tomato plant with wilting symptoms were purified and observed under an electron microscope [22]. Thus, the purified virus allowed the RNA extraction and subsequent genomic characterization. Once the ToMarV sequence was known, it allowed the design of assays based on RT-PCR for detection [98]. Additionally, Western and Northern blot tests were reported during the production of an infectious clone of ToMarV [97].

For the management of the disease caused by ToMarV, it is key to eliminate the infected plants, control whitefly populations, and eliminate the host weeds inside and near the production areas. Consequently, the early detection of the virus by molecular assays is important to prevent an outbreak.

## 7. Conclusions and Perspectives

Year-to-year tomato production can be affected by different diseases caused by viruses such as those described in this review. The diseases of higher economic impact are those induced by viruses that are transmitted by seed and that also have the particularity of being mechanically transmitted, as in the case of ToBRFV and PepMV, which are rapidly disseminated around the world and have caused damages with significant economic losses, principally when crops are affected in their early stages of development. On the other hand, viruses that are transmitted by insects, such as TSWV, TYLCV, and ToMarV, are found in specific areas, and their handling depends in great measure on the approaches to control the vector.

This review provides relevant and updated information regarding the major viruses that cause severe economic losses in tomato crops in Mexico and also provides information on other viruses that have been determined to cause diseases of minor impact. However, other viruses that have caused critical issues and epidemics in tomato in other countries could potentially threaten production in Mexico, and knowledge should be acquired for identification and management if these were to become introduced. Among the viruses that present a potential threat for tomato production in Mexico, there can be found some members of the genera *Alphanucleorhabdovirus* (Physostegia chlorotic mottle alphanucleorhabdovirus), *Begomovirus* (tomato severe rugose virus, tomato yellow vein streak virus, tomato rugose yellow leaf curl virus, tomato chlorotic leaf curl virus, tomato chlorotic leaf distortion virus, and tomato dwarf leaf virus), *Blunervirus* (tomato fruit blotch virus), *Ilarvirus* (tomato necrotic streak virus, tobacco streak virus, and Parietaria mottle virus), *Orthotospovirus* (tomato chlorotic spot virus, groundnut ringspot virus, tomato yellow ring virus, tomato zonate spot virus, Alstroemeria necrotic streak virus, and Capsicum chlorosis virus), *Potyvirus* (chilli veinal mottle virus), *Tobamovirus* (tobacco mild green mosaic virus), *Topilevirus* (tomato apical leaf curl virus), *Torradovirus* (tomato torrado virus), and *Tymovirus* (tomato blistering mosaic tymovirus), among others.

## Figures and Tables

**Table 1:** Viruses reported on tomato crops in Mexico.

Virus	Abrev.	Family	Genus	Reported Location	References
Chino del tomate virus	CdTV	*Geminiviridae*	*Begomovirus*	Sinaloa, Sonora	[3,4]
Pepper golden mosaic virus	PepGMV	*Geminiviridae*	*Begomovirus*	Sinaloa, Baja California Sur, Nayarit, Hidalgo	[5,6,7]
Sinaloa tomato leaf curl virus	STLCV	*Geminiviridae*	*Begomovirus*	Sinaloa	[8]
Tomato mottle virus	ToMoV	*Geminiviridae*	*Begomovirus*	Yucatán	[9]
Pepper huasteco yellow vein virus	PHYVV	*Geminiviridae*	*Begomovirus*	Jalisco, Morelos, San Luis Potosí, Hidalgo	[4,7]
Tomato yellow leaf curl virus	TYLCV	*Geminiviridae*	*Begomovirus*	Sinaloa, Sonora, Yucatán	[10,11,12]
Tomato chino la paz virus	ToChLPV	*Geminiviridae*	*Begomovirus*	Baja California Sur	[13]
Tomato severe leaf curl virus	ToSLCV	*Geminiviridae*	*Begomovirus*	Estado de México, San Luis Potosí	[14,15]
Tomato golden mottle virus	ToGMoV	*Geminiviridae*	*Begomovirus*	San Luis Potosí	[16]
Squash leaf curl virus	SLCV	*Geminiviridae*	*Begomovirus*	Sinaloa	[12]
Tomato mosaic virus	ToMV	*Virgaviridae*	*Tobamovirus*	Estado de México, Jalisco	[7,15]
Tomato mottle mosaic virus	ToMMV	*Virgaviridae*	*Tobamovirus*	Jalisco	[17]
Tomato brown rugose fruit virus	ToBRFV	*Virgaviridae*	*Tobamovirus*	Michoacán, Baja California Sur	[18,19]
Tomato ringspot virus	ToRSV	*Secoviridae*	*Nepovirus*	Guanajuato	[20]
Tomato marchitez virus	ToMarV	*Secoviridae*	*Torradovirus*	Sinaloa	[21,22,23]
Tobacco etch virus	TEV	*Potyviridae*	*Potyvirus*	Oaxaca	[7]
Tomato necrotic stunt virus	ToNStV	*Potyviridae*	*Potyvirus*	Near Mexico City	[24]
Tomato chlorosis virus	ToCV	*Closteroviridae*	*Crinivirus*	Sinaloa	[25]
Tomato infectious chlorosis virus	TICV	*Closteroviridae*	*Crinivirus*	Baja California	[26]
Tomato spotted wilt virus	TSWV	*Tospoviridae*	*Orthotospovirus*	Puebla, Morelos, Estado de México, Sinaloa, Guanajuato, Baja California	[20,27,28]
Impatiens necrotic spot virus	INSV	*Tospoviridae*	*Orthotospovirus*	Estado de México	[15]
Southern tomato virus	STV	*Amalgaviridae*	*Amalgavirus*	Colima	[29]
Pepino mosaic virus	PepMV	*Alphaflexiviridae*	*Potexvirus*	Estado de México	[15,30]
Cucumber mosaic virus	CMV	*Bromoviridae*	*Cucumovirus*	Colima	[31]

## Data Availability

Not applicable.
